# A Form of Gaseous Gangrene: With Special Reference to Malignant Gaseous Edema

**Published:** 1917-12

**Authors:** 


### GAS GANGRENE

### ETIOLOGY

***A Form of Gaseous Gangrene : with Special Reference to Malignant Gaseous Edema.*** Based upon work done by M. Sacquépée : reported at a Meeting of the *Société de Chirurgie* by E. Quenu. *Bulletins et Mémoires de la Société de Chirurgie de Paris*, June 1, 1915.

The form of gaseous gangrene described in this article is what S. terms " malignant gaseous edema ". The clinical characteristics are : a considerable edema of the limb, generally more marked at the extremity than near the body; a bronze discoloration at one point but paleness elsewhere ; an abrupt demarcation of the swelling as in erysipelas. The neighboring muscles as well as the subcutaneous tissues are infiltrated. The discharge from an incision in the skin near the wound appears brown, but slightly yellowish or colorless from an incision at a little distance. An emphysema of a much less extent appears at the same time, and is sometimes imperceptible clinically, affecting only those tissues, in the vicinity of the wound. Incisions show no gas to be present in the region of the edema. For this reason S. considers the edema the principal symptom, and the emphysema of secondary importance. He notes the peculiar odor of putrefaction, that escapes from the lesions and the infrequent appearance of blebs on the skin. The general condition is caracterized by dyspnea without any considerable pulmonary lesions, cooling of the body, weak, high pulse, and a pale, thin yellowish, complexion. The mind remains clear.

In a few hours the gaseous edema develops, and when left to itself, invariably proves fatal. It may continue for twelve hours or up to four days. The general and local phenomena do not correspond necessarily in their development, for, although the general phenomena appear together with local ones, they may become acute, even resulting in death, while the local phenomena remain limited, rarely involving the whole of a single member.

From a careful anatomical study S. concludes that *at the start the lesion is entirely muscular*. (The Italics are the editors'.) There is usually found at a definite point in the muscular mass near the wound, although not, necessarily contiguous, a gangrenous center, sometimes the size of a fist, appearing as a homogeneous necrosed mass easily crushed; at a less advanced stage, this center may be no larger than a thumb, and may be hidden away in the muscle so as to escape detection. Gases escape from this mass, but they often remain for a considerable time in the muscle before reaching the subcutaneous tissue.

By bacteriological examination S. has established the presence of a bacillus thicker than the Vibrion septique, straight or slightly curved, and anaerobic. It is to be found at the gangrenous center or in the serosity near it, but rarely in the muscles, in the peripheral blood, or in the serosity of the edema. With this bacillus S. has reproduced in the guinea-pig the same phenomena as are to be observed in man. S. therefore concludes that the bacillus is specific and differs from the Vibrion septique and the perfringens.

Q. appends the detailed reports of two cases.

In the *discussion* of Q.'s paper M. le Dentu called the attention of the Society to what appeared to him to be two distinct types of gaseous gangrene, one of which he characterized as " edema almost entirely without gas " and the other as " a development of gases entirely or almost entirely independent of the symptoms of gangrene or even of edema ".

In reply to a question put by M. Tuffier, as to whether gas gangrene appeared to S. to be superficial or shallow, Q. deplored the confusion that had arisen by grouping various phenomena, which have neither the same traits nor the same development as classic gas gangrene, under this heading as a matter of convenience. Q. therefore believed that the work undertaken by S. was especially valuable because it tended to limit the conception of gas gangrene to a specific pathogenic type. Not all gas discharges indicated gas gangrene, even in cases where other attributes of gangrene were present.

M. Tuffier then stated that he believed it necessary to distinguish not only *various forms* of gangrene, but *anatomical varieties* as well. Possibly the superficial sub-cutaneous variety was not so serious as the deeper infections. The speaker had noted that intra-muscular infections were far more difficult to treat than the more superficial varieties. Injections of oxygen gas which sufficed to check the infection in cases of " superficial gas gangrene " were powerless against the intra-muscular variety.

